# The effect of sperm DNA fragmentation on *in vitro* fertilization outcomes of unexplained infertility

**DOI:** 10.1016/j.clinsp.2023.100261

**Published:** 2023-07-27

**Authors:** Qingxin Wang, Xiaoling Gu, Yannan Chen, Minyan Yu, Lingna Peng, Shuping Zhong, Xia Wang, Jinxing Lv

**Affiliations:** a Dushu Lake Hospital Affiliated to Soochow University, Jiangsu, China; b Affiliated Hospital of Nantong University, Jiangsu, China; c The First Affiliated Hospital of Soochow University, Jiangsu, China

**Keywords:** Unexplained infertility, Sperm DNA fragmentation index, D3 good-quality embryos, Live birth rate

## Abstract

**Background:**

Infertility is caused by heterogeneous risks, but most of them are unexplained. The sperm DNA Fragmentation Index (DFI) was increasingly acknowledged as a parameter for the evaluation of male infertility. This study aimed to investigate the association between sperm DFI and laboratory and clinical outcomes in a population with unexplained infertility.

**Methods:**

The clinical data of an infertile population was collected for the selection of reproductive patients with unexplained infertility. The authors classified the patients with normal sperm parameters in a control group (DFI < 25%) and an observation group (DFI ≥ 25%) and compared the difference in basal characteristics, laboratory, and clinical outcomes between the two groups. The authors conducted a correlation analysis to examine the relationship between DFI and the number of D3 good-quality embryos, as well as the clinical pregnancy rate and live birth rate. A total of 176 cases were enrolled in the retrospective study.

**Results:**

The observation group (n = 88) showed advanced male age, lower sperm concentration, progressive motility, and morphology assessment than the control group. In addition, lower No. of D3 good-quality embryos, clinical pregnancy rate, and the live birth rate were shown in the observation group. A negative correlation between the DFI and No. of D3 good-quality embryos (rs = -0.347, p < 0.001) or live birth rate (rs = -0.185, p = 0.028) was shown.

**Conclusions:**

Sperm DFI was a good indicator for the prediction of D3 good-quality embryos in unexplained infertility couples, but it did not provide sufficient information regarding clinical pregnancy outcome but live pregnancy outcome.

## Highlights

• Infertility is caused by heterogeneous risks, but most of them are unexplained.
• Sperm DFI diagnostic value was controversial as not rule out male sperm parameters.
• Whether DFI was a useful indicator for embryologists and clinicians in UEI couples.

## Introduction

Unexplained Infertility (UEI) refers to the inability to conceive despite 12 months of unprotected intercourse where known causes of infertility have been ruled out. The contributing factors for infertility are complex and often involve a combination of male and female factors. While male and female factors account for approximately 1/3 of each of infertility cases, the remaining 1/3 of couples are diagnosed with unexplained infertility, despite current diagnostic assessments and traditional semen analysis.^1^ The conception of UEI was first proposed in the 1960s; however, effective treatments have yet to be developed.^2^ Currently, expectant management is the preferred method for treating UEI.^3^ Nonetheless, it has been suggested that positive interventions such as pharmacological approaches or Assisted Reproductive Technologies (ART) could improve clinical outcomes.^4^ ART as an effective tool, identify some of the causative factors underlying UEI, such as sperm or egg abnormalities that prevent fertilization or the formation of high-quality embryos or blastocysts during cleavage stages, which current clinical examinations are unable to detect.

The use of the Sperm DNA Fragmentation Index (DFI) has been proposed as a means of measuring sperm DNA damage in clinical practice. However, the diagnostic value of sperm DFI has been subject to debate and remains controversial, particularly given that major studies focused on couples affected by infertility, which can have complex and multifactorial etiological factors.^5^^,^^6^ Despite efforts to address confounding factors by setting up a series of inclusion and exclusion criteria, it is difficult to eliminate their effects completely. Recent guidelines have highlighted the clinical utility of sperm DFI, particularly in cases of 'unexplained infertility.^7^^,^^8^ Studies have indicated that men diagnosed with unexplained infertility tend to have elevated DFI levels.^9^ Regarding the outcome of individuals with high DFI, certain studies have indicated a decrease in the pregnancy rate as well as the rate of high-quality embryo production following In Vitro Fertilization (IVF).^10^ Conversely, other studies have not found a significant correlation in cases where Intracytoplasmic Sperm Injection (ICSI) is utilized.^11^

Our study is aimed to investigate whether DFI was a useful indicator for embryologists and clinicians in UEI couples, to examine the possible diagnostic criteria of sperm DFI for laboratory and clinical outcomes, and to provide a more comprehensive guide for clinical practice.

## Materials and methods

### Patient selection

**Fig. 1:**
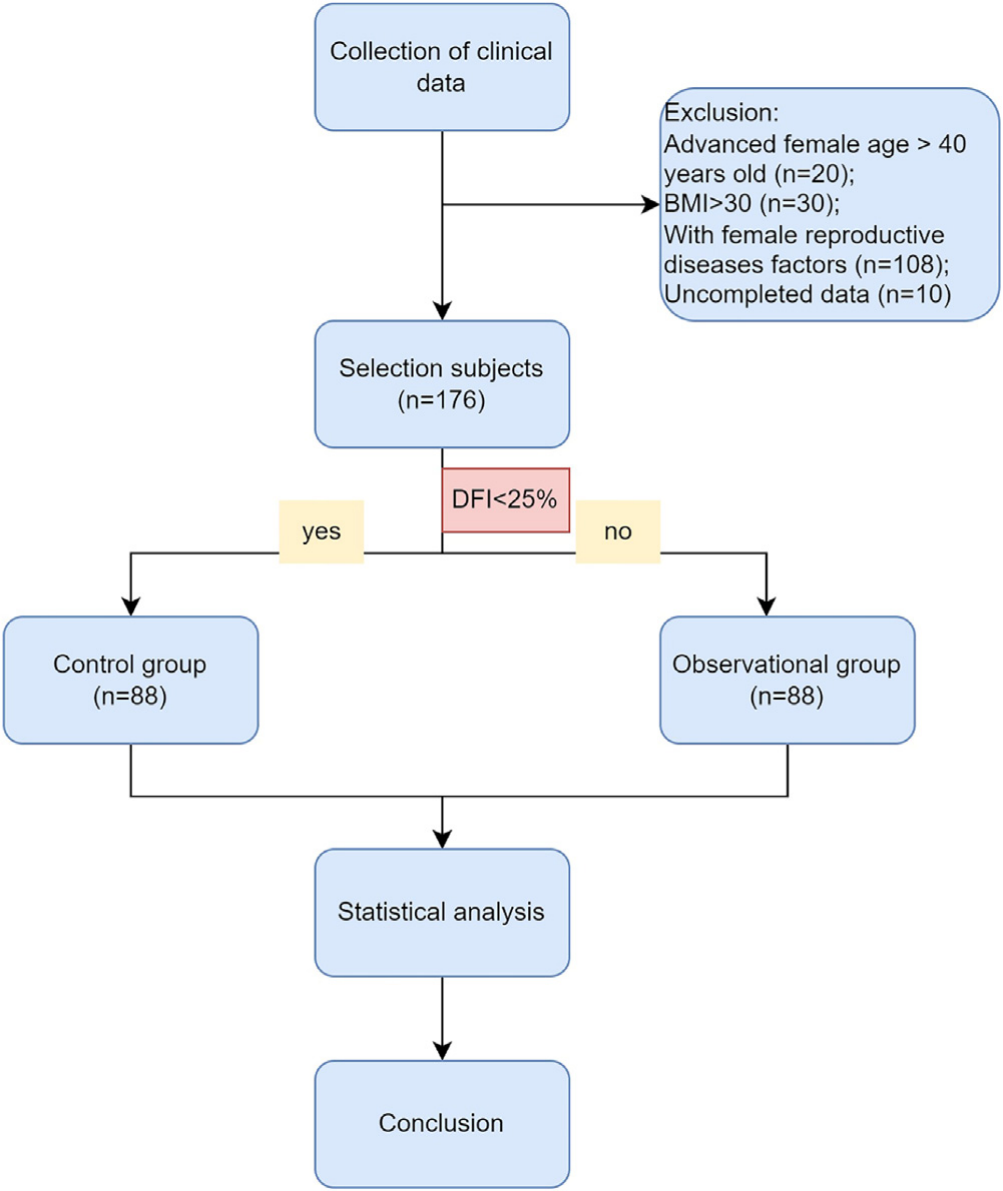
Flow chart of the retrospective study.

The authors conducted a retrospective analysis of data from 176 couples with unexplained infertility at the Affiliated Hospital of Nantong University. From January 1, 2017, to March 31, 2022, we used the following inclusion criteria for the diagnosis of unexplained infertility: 1) Failure to conceive after one year or more of regular, unprotected intercourse with the same partner; 2) Male age below 45-years old and normal sperm parameters according to WHO, 2010 (concentration, motility, and morphology) and no andrological history of concern (cryptorchidism, hypogonadotropic hypogonadism, genetic abnormalities like Klinefelter's syndrome or Y-chromosome microdeletion, drug abuse, cancer treatment, or other iatrogenic factors); 3) No female factors including advanced age, low body weight or overweight, anovulation, adenomyosis, tubal factor, chromosome abnormality, pelvic inflammation, uterine fibroid, and uterine malformation, which may adversely affect clinical outcomes. The exclusion criteria were shown as.Figure 1, including advanced female age over 40 years old, female BMI over 30, and uncompleted data.

This study was approved by the Ethics Committee of the Affiliated Hospital of Nantong University (2022-K076-01) and followed the STROBE Statement. Informed consent was exempted in accordance with the urgent situation and the Ethics Committee's rules.

### Semen analysis and DNA fragmentation assay

All men underwent a routine semen analysis and Sperm Chromatin Dispersion (SCD) test one month prior to the IVF procedure. According to the World Health Organization guidelines (WHO, 2010), conventional semen analysis (sperm concentration and motility) was carried out using a Computer-Assisted Sperm Analyzer (Weili, Beijing, China) and morphology by staining with the Papanicolaou method.

SCD test (ShenZhen Huakang CO., LTD, China) was carried out according to the manufacturer's instructions as described in our previous study.^12^

### IVF/ICSI procedures

The authors used controlled ovarian stimulation, using a combination of a gonadotropin-releasing hormone agonist, a gonadotropin-releasing hormone antagonist, and recombinant follicle-stimulating hormone/human menopausal gonadotropin. Once the three dominant follicles reached a mean diameter of at least 17 mm, the authors injected 250 μg of recombinant human chorionic gonadotropin (hCG; Ovidrel, Serono) and retrieved oocytes using transvaginal ultrasound guidance 36 hours later. The authors then incubated the oocytes in G-IVFTM (Vitrolife, Gothenburg, Sweden) medium supplemented with 10% human serum albumin (Vitrolife) and performed In Vitro Fertilization (IVF) or Intracytoplasmic Sperm Injection (ICSI) 4‒6 hours after retrieval. Normal fertilization was determined by the presence of two pronuclei 16‒20 hours after insemination. Embryonic development was monitored at 48 and 72 hours after retrieval and graded at 72 hours based on the number of cells, level of fragmentation, and cell size variation.^13^ High-quality embryos were defined as grade I and II and reserved for later embryo transfer. The authors only included the first Embryo-Transfer (ET) cycles of fresh or thawed embryos.

### Primary outcomes assessment

The study examined the number of Two Pronuclei (2PN) (which indicated a normal fertilized zygote or embryo), identified the day after In Vitro Fertilization (IVF). The number of cleaved embryos with two or more blastomeres was defined two days after IVF. The embryo quality grading was determined on the day of embryo transfer (day 3 or 5) and divided into good quality (grade 1 and 2) and poor quality (grade 3 and 4).^14^ Biochemical pregnancy was defined as a positive test of human chorionic gonadotropin in the absence of any ultrasonographic evidence of pregnancy, and no evidence or treatment of an extra uterine pregnancy. Clinical pregnancy was identified as an intrauterine pregnancy with fetal cardiac activity confirmed by transvaginal ultrasound at 7 weeks’ gestation. The biochemical pregnancy rate per started cycle was the percentage of cases with biochemical pregnancy out of the total cases that received embryo transfer. A twin pregnancy was diagnosed by a senior doctor using ultrasound. First-trimester miscarriage rate = the percentage of nonviable clinical pregnancy/clinical pregnancy, noted by ultrasound follow-up until gestational week 12 of pregnancy. Biochemical pregnancy rate per started cycle = the number of cases with biochemical pregnancy / the total number of cases with embryo transfer per started cycle × 100%. Clinical pregnancy rate per started cycle = the number of cases with clinical pregnancy / the total number of cases with embryo transfer per started cycle × 100%. Twin pregnancy rate per started cycle = the number of cases with twin pregnancy / the total number of cases with embryo transfer per started cycle × 100%. First-trimester miscarriage per started cycle = the number of cases with miscarriage before gestational week 12 of pregnancy / the total number of cases with embryo transfer per started cycle × 100%. Live birth rate per started cycle = the number of cases with miscarriage / the total number of cases with embryo transfer per started cycle × 100%.

### Secondary outcomes assessment

In this study, relevant information including the age and Body Mass Index (BMI) of participating couples were collected. Female basal Follicle Stimulating Hormone (FSH), Luteinizing Hormone (LH), and 17β-Estradiol (E2) levels were tested using kits in accordance with the guidelines provided by the manufacturer (Sangon Biotech, China). Following recombinant hCG injection, LH and E2 levels were retested. Oocyte retrieval surgery was conducted by experienced senior surgeons, and prior to embryo implantation, endometrial thickness was measured by a specialist in ultrasound.

### Statistical analysis

Statistical analysis was performed using the SPSS statistical package (version 26.0, SPSS Inc., Chicago, IL, USA). The normality of continuous variables was analyzed by a Shapiro-Wilk test. The standard normally distributed data are described as the mean ± Standard Deviation (SD) and were compared by a Student's *t*-test. Nonnormally distributed variables are expressed as the median (interquartile range) and were compared by a Mann-Whitney *U* test. Categorical variables were described as concrete cases (percentages) and compared by a Chi-Square test or Fisher's exact test. Spearman's correlation analysis was used if the two continuous variables were not normally distributed; otherwise, Kendall's tau-b was used for correlation analysis if there were categorical variables. A value of p<0.05 was considered statistically significant.

## Results

### The comparison of male and female characteristics between the two groups

A total of 344 infertile couples were initially enrolled in this study. Following the exclusion criteria outlined in Fig. 1, 176 subjects were ultimately selected for inclusion. Participants were then categorized into two groups based on their DNA Fragmentation Index (DFI) value: a control group (DFI <25%, n = 88) and an observational group (DFI ≥ 25%, n = 88). The median DFI values in the observational and control groups were 33.8 and 10, respectively. Significant differences were observed between the two groups in terms of several female characteristics, including age, BMI, basal FSH, E_2_, LH levels, and E_2_/LH levels on hCG day. However, there were no significant differences in the number of oocytes or MII oocytes retrieved. Male age was found to be older in the observational group compared to the control group. In addition, while sperm concentration, progressive motility, and morphology were within normal ranges for both groups, poorer sperm quality was observed in the group with higher DFI. These findings suggest that ICSI may offer greater potential for fertilization success in cases where a higher DFI is present (Table 1).

**Table 1:** Baseline characteristics comparison between control group (DFI <25%) and observation group (DFI ≥ 25%).

Variable	Control group (n = 88)	Observation group (n = 88)	χ^2^/U value	p-value
Female age (years)	28 (26∼30)	30 (28∼35)	5339.5	<0.001
Female BMI (kg/m^2^)	21 (19∼23)	22.5 (20.6∼24.5)	4980.5	0.001
Basal FSH levels (IU/L)	6.5 (5.3∼7.4)	6.9 (5.9∼8.5)	4710.5	0.013
Basal LH levels (IU/L)	4.3 (3.4∼5.8)	4.2 (2.8∼5.8)	3534.5	0.318
Basal E_2_ levels (pg/mL)	46.4 (39.3∼57.3)	37.0 (27.0∼52.7)	2547.0	<0.001
Total gonadotropin dose (IU)	1725.0 (1500.0∼2006.3)	1800.0 (1425.0∼2081.3)	3963.0	0.787
No. of days of stimulation	7 (7∼8)	8 (7∼8)	4255.5	0.242
E2 of hCG day (pg/mL)	2840.0 (2026.1∼3560.0)	2240.0 (1246.0∼3936.4)	3173.0	0.039
LH of hCG day (IU/L)	1.5 (1.0∼2.5)	2.4 (1.2∼3.8)	4988.5	<0.001
P of hCG day (ng/mL)	1.1 (0.8∼1.4)	0.9 (0.6∼1.4)	3344.0	0.117
Endometrial thickness (mm)	10.0 (8.9∼12.4)	10.6 (8.5∼12.0)	3739.0	0.694
No. of oocytes retrieved	9 (7∼14)	8 (5∼12)	3241.5	0.061
MII oocytes retrieved rate, (%)	100.0 (50.0∼100.0)	93.7 (50.0∼100.0)	3605.5	0.386
Male age (years)	29 (27∼31)	33 (29∼37)	5503.0	<0.001
Male BMI (kg/m^2^)	23.6 (20.8∼25.9)	24.2 (22.1∼26.0)	4372.5	0.138
Infertility duration (yr)	3 (2∼5)	4 (2∼5)	4164.5	0.384
Sperm concentration (*10^6^/mL)	64.0 (45.1∼89.2)	46.8 (28.1∼75.5)	2890.0	0.004
Progressive motility (%)	46.0 (40.0∼53.8)	37.5 (32.0∼48.0)	2272.5	<0.001
Sperm morphology (%)	6.0 (5.0∼7.0)	5.0 (4.0∼6.0)	2918.0	0.004
Sperm DFI (%)	10.0 (6.1∼14.0)	33.8 (29.3∼41.0)	7744.0	0.000
ART			42.764	<0.001
IVF	76 (86.4)	34 (38.6)		
ICSI	12 (13.6)	54 (61.4)		

#### Lower good-quality embryo rates, clinical pregnancy rates and live birth rates in the cases with higher DFI

**Fig. 2:**
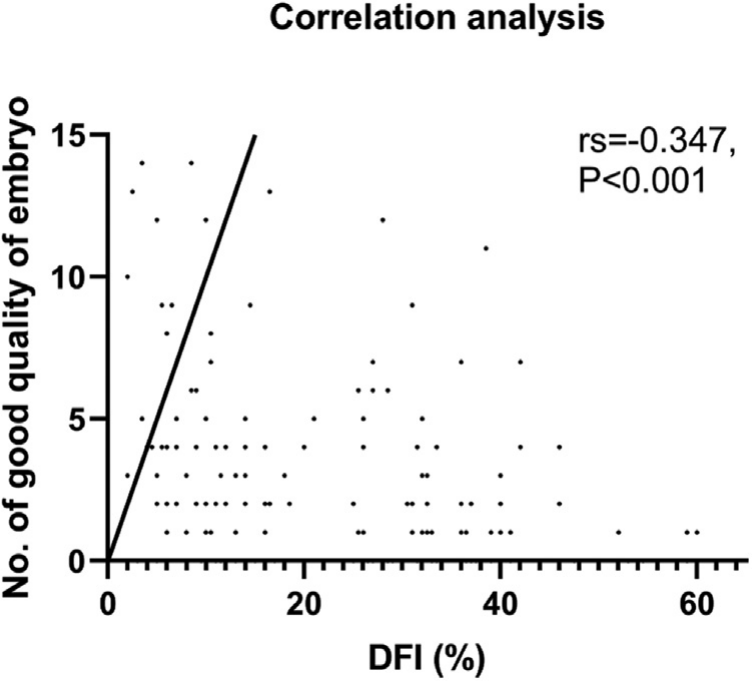
Correlation analysis between DFI and No. of D3 good quality embryo.

Our analysis found no significant differences between the two groups in terms of the number of 2PN embryos or normal fertilized and cleavage embryo rates. Although, it was observed that the number of good-quality embryos was lower in the observation group. A negative correlation was found between DFI and the number of good-quality embryos, which was statistically significant (rs = -0.347, p < 0.001, Fig. 2). As the control group had more viable embryos, they were able to implant two embryos, unlike the observation group which had fewer. It is worth noting, however, that no significant differences were observed in the implantation of frozen versus fresh embryos between the two groups. Nevertheless, the clinical results revealed that the observation group had lower clinical pregnancy and live birth rates per cycle, which could be attributed to the lower quality of embryos due to increased DFI. After further correlation analysis, it was found that only the live birth rate per cycle was negatively correlated with DFI (rs = -0.185, p < 0.028), while there was no such relationship with the clinical pregnancy rate (p = 0.123) (Table 2). It is noteworthy that there were no differences in biochemical pregnancy rates, twin pregnancy rates, or miscarriage rates per cycle between the two groups (Table 3).

**Table 2:** Correlation analysis between clinical pregnancy rate or live birth rate per started cycle and DFI.

Variables	rs	p-value
Clinical pregnancy rate per started cycle	-0.130	0.123
Live birth rate per started cycle	-0.185	0.028

**Table 3:** Clinical outcome comparison between control group (DFI <25%) and observation group (DFI ≥ 25%).

	Control group (n = 88)	Observation group (n = 88)	χ^2^/U value	p-value
No. of 2PN embryo	4.5 (2.3∼7.0)	4.0 (2.0∼6.0)	3466.5	0.228
Normal fertilized embryo rate (%)	75.0 (51.8∼97.7)	70.3 (50.0∼91.7)	3697.5	0.154
No. of Cleavage embryo	4.5 (2.3∼7.0)	4.0 (2.0∼6.0)	3393.5	0.154
Cleavage embryo rate (%)	100.0 (100.0∼100.0)	100.0 (100.0∼100.0)	3507.0	0.113
No. of good quality embryo	2.5 (1.0∼4.8)	1.0 (0.0∼2.0)	2581.0	<0.001
Good quality embryo rate (%)	58.6 (28.6∼92.7)	25.0 (0.0∼62.4)	2744.5	<0.001
Number of embryo implantation				
1, n (%)	12 (18.5)	17 (53.1)	12.293	<0.001
2, n (%)	53 (81.5)	15 (46.9)		
Frozen fertilized embryo implantation, n (%)	55 (84.6)	26 (81.3)	0.176	0.675
Biochemical pregnancy rate per started cycle, n (%)	6 (9.2)	5 (15.6)	0.872	0.350
Clinical pregnancy rate per started cycle, n (%)	37 (56.9)	9 (28.1)	7.132	0.008
Twin pregnancy rate per started cycle, n (%)	9 (14.3)	4 (12.9)	0.033	0.855
Miscarriages per started cycle, n (%)	6 (9.2)	3 (9.4)	0.001	0.982
Live birth rate per started cycle, n (%)	28 (43.1)	4 (12.5)	9.069	0.003

### Correlation analysis of potential influence factors on the outcome of D3 good quality embryo

To gain a better understanding of the potential factors that could affect the number of good-quality embryos, the authors conducted a correlation analysis between significant variables and the aforementioned number. Our findings revealed that there exists a negative correlation between female age (rs = -0.170, p = 0.024), BMI (rs = -0.152, p = 0.044), as well as male age (rs = -0.212, p = 0.005) with the number of good quality embryos. Conversely, the authors discovered that basal E2 levels (rs = 0.190, p = 0.011), as well as sperm progressive motility (rs = 0.255, p < 0.001) and morphology (rs = 0.179, p = 0.018) all presented a positive correlation with the number of good quality embryos (Table 4).

**Table 4:** Correlation analysis of potential influence factors on the outcome of D3 good quality embryo.

Variables	rs	p-value
Female age	-0.17	0.024
Female BMI	-0.152	0.044
Basal E2 levels	0.190	0.011
LH of hCG day	-0.085	0.263
Male age	-0.212	0.005
Sperm concentration	0.016	0.828
Progressive motility	0.255	<0.001
Sperm morphology	0.179	0.018
ART	-0.045	0.494

## Discussion

In our findings, it has been observed that the age of the male participants is higher, and their sperm DNA Fragmentation Index (DFI) is greater. This result is in agreement with the previous literature on the subject.^15^^,^^16^ It is widely understood that the majority of these scenarios have a direct correlation with the elevated production of Reactive Oxygen Species (ROS), which have been found to be destructive to sperm DNA and result in fragmentation upon entering the cell nucleus. Additionally, ROS has a profound effect on sperm motility.^17^

Additionally, our findings indicate that under normal conditions of sperm semen, there is a correlation between the DFI and decreased sperm concentration, progressive motility, and sperm morphology. Sperm DNA is crucial for successful embryonic development and can have a significant impact on the chances of both natural and assisted pregnancy.^18^ However, traditional sperm analysis methods have certain limitations. To address this issue, sperm DFI, which measures the proportion of sperm with damaged DNA in semen, has emerged as a promising new tool for sperm assessment.^15^

A number of studies have proposed that DNA Fragmentation Index (DFI) in sperm has a negative impact on fertilization rates.^19^ Furthermore, research has suggested that there is a lower percentage of good-quality embryos in the high DNA damage group, and embryos from males with high DFI are more difficult to implant,^20^^,^^21^ leading to pregnancy loss^22^ However, other studies have found no significant differences in rates of clinical pregnancy, early abortion, oocyte fertilization, or good-quality embryos.^23^^,^^24^ These contrasting results may be due to differences in assessment methods, thresholds, reagents, sample size, and inclusion criteria for participants. Additionally, most studies have not ruled out the effects of sperm-related factors, such as motility, including our previous research.^12^ Therefore, it remains unclear whether it is sperm motility or DFI that affects ART outcomes. Our results indicate that there are lower live birth rates (rs = -0.185) in the population with a higher DFI. However, there was no relationship found between DFI and clinical pregnancy rate, despite a significant difference being present between the high DFI and control groups. The possible reason for such a result could be attributed to the quality of the embryo. Our analysis of couples with unexplained infertility showed a negative correlation between sperm DFI values and good-quality embryos (rs = -0.347), which was stronger than other potential factors such as female age (rs = -0.170), BMI (rs = -0.152), male age (rs = -0.212), basal E2 levels (rs = 0.190), sperm progressive motility (rs = 0.255), and morphology (rs = 0.179). The lower live birth rate in the high DFI group might be resulted from poor embryo quality in this group. One meta-analysis of eight studies comprising 17,879 embryos revealed a lower good-quality embryo rate with a higher DFI (RR = 0.65 [0.62, 0.68]. p < 0.01).^10^

However, our study is subject to several limitations that should be acknowledged. Firstly, the sample size was small, which may have limited the statistical power of our analysis. Additionally, the authors only used one clinical testing method, SCD, to detect sperm DFI. While this method is simple and affordable, it is more susceptible to subjective factors and may not accurately reflect the complete status of sperm DNA.^25^^,^^26^ Different techniques may yield different results and show different aspects of sperm DNA status,^27^ making it difficult to compare and correlate DFI values from each method.^26^ Furthermore, our study only examined the first embryo-transfer cycles in which the authors selected the best-quality embryos for transfer, which may have introduced selection bias in our analysis of clinical outcomes. As a result, the cumulative pregnancy rate could not be calculated, which would have provided more valuable information given the differences observed in high-quality embryo rates. This limitation is common in studies examining the impact of statistics on clinical outcomes and highlights the need for further research.

## Conclusion

The Sperm DFI proved to be a useful predictor of high-quality D3 embryos for couples facing unexplained infertility. However, it may not provide adequate insights into the clinical pregnancy outcome, and instead, a live pregnancy outcome is a more informative metric. A refined approach to interpreting Sperm DFI findings can, therefore, benefit fertility treatment outcomes for such couples.

## Ethics approval and consent to participate

This study was approved by the Ethics Committee of the Affiliated Hospital of Nantong University (2022-K076-01). Informed consent was exempted in accordance with the urgent situation and the Ethics Committee's rules.

## CRediT authorship contribution statement

**Qingxin Wang:** Data curation, Project administration, Investigation, Funding acquisition, Methodology, Writing – original draft, Writing – review & editing. **Xiaoling Gu:** Data curation. **Yannan Chen:** Data curation. **Minyan Yu:** Data curation. **Lingna Peng:** Data curation. **Shuping Zhong:** Data curation. **Xia Wang:** Data curation, Project administration, Investigation, Writing – review & editing. **Jinxing Lv:** Project administration, Investigation, Funding acquisition, Writing – review & editing.

## Declaration of Competing Interest

The authors declare no conflicts of interest.
